# KansformerEPI: a deep learning framework integrating KAN and transformer for predicting enhancer–promoter interactions

**DOI:** 10.1093/bib/bbaf272

**Published:** 2025-06-14

**Authors:** Tianjiao Zhang, Saihong Shao, Hongfei Zhang, Zhongqian Zhao, Xingjie Zhao, Xiang Zhang, Zhenxing Wang, Guohua Wang

**Affiliations:** College of Computer and Control Engineering, Northeast Forestry University, No. 26 Hexing Road, Xiangfang District, Harbin 150040, China; College of Computer and Control Engineering, Northeast Forestry University, No. 26 Hexing Road, Xiangfang District, Harbin 150040, China; College of Computer and Control Engineering, Northeast Forestry University, No. 26 Hexing Road, Xiangfang District, Harbin 150040, China; College of Computer and Control Engineering, Northeast Forestry University, No. 26 Hexing Road, Xiangfang District, Harbin 150040, China; College of Computer and Control Engineering, Northeast Forestry University, No. 26 Hexing Road, Xiangfang District, Harbin 150040, China; College of Computer and Control Engineering, Northeast Forestry University, No. 26 Hexing Road, Xiangfang District, Harbin 150040, China; College of Computer and Control Engineering, Northeast Forestry University, No. 26 Hexing Road, Xiangfang District, Harbin 150040, China; College of Computer and Control Engineering, Northeast Forestry University, No. 26 Hexing Road, Xiangfang District, Harbin 150040, China; Faculty of Computing, Harbin Institute of Technology, No. 92 West Da Zhi street, Nangang District, Harbin 150001, China

**Keywords:** enhancer–promoter interactions, KAN, transformer, deep learning

## Abstract

Enhancer–promoter interaction (EPI) is a critical component of gene regulation. Accurately predicting EPIs across diverse cell types can advance our understanding of the molecular mechanisms behind transcriptional regulation and provide valuable insights into the onset and progression of related diseases. At present, large-scale genome-wide EPI predictions typically rely on computational approaches. However, most of these methods focus on predicting EPIs within a single cell line and lack a global perspective encompassing multiple cell lines. Furthermore, they often fail to fully account for the nonlinear relationships between features, leading to suboptimal prediction accuracy. In this study, we propose KansformerEPI, a global EPI prediction model designed for multiple cell lines. The model is built on Kansformer, an encoder that integrates KAN and Transformer, effectively capturing the nonlinear relationships among various epigenetic and sequence features. We utilized KansformerEPI to achieve cross-tissue prediction of EPIs across different cell types. This approach enhances the model’s scalability, eliminating the complexity of designing separate prediction models for individual tissues. As a result, our model is applicable to various tissues, thereby reducing dependency on extensive datasets. Experimental results demonstrate that KansformerEPI surpasses existing methods such as TransEPI, TargetFinder, and SPEID in both accuracy and stability of EPI predictions across datasets including HMEC, IMR90, K562, and NHEK.

## Introduction

Enhancers and promoters are two critical DNA sequence elements in cells. Enhancers play an essential role in the transcriptional regulation of genes, significantly increasing the expression of target genes [1, 2]. Promoters, often referred to as the “switches” of gene expression, are typically defined as regions spanning 1500 bp upstream to 500 bp downstream of the transcription start site (TSS) [3, 4]. As *cis*-regulatory elements in the genome, enhancers regulate gene expression through interactions with their target gene promoters [5, 6]. Recent studies on the 3D genome [7] have revealed that distal enhancers can physically interact with proximal promoters to regulate target gene expression. Accurate identification of enhancer–promoter interactions (EPIs) is vital for understanding gene regulation, cellular differentiation, and disease mechanisms[8–10]. Notably, some variations within enhancer regions have been linked to cancer susceptibility. For example, the 8q24 locus contains multiple functional enhancers associated with various tumor types [11]. However, due to the often considerable genomic distances between enhancers and their target promoters, accurately identifying EPIs remains a significant challenge.

Previous studies have inferred EPIs using expression quantitative trait loci (eQTL) mapping [12, 13]. However, eQTL mapping typically requires large sample sizes and primarily identifies short-range EPIs [14, 15]. With the advent of high-throughput sequencing technologies, researchers can now leverage techniques such as chromatin interaction analysis with paired-end tag sequencing (ChIA-PET [16]) and high-throughput chromosome conformation capture (Hi-C [17]) to detect long-range chromatin interactions, which can be used to identify EPIs. For instance, the application of Hi-C has revealed multiple topologically associated domains (TADs), with studies indicating that interaction frequencies between chromatin regions are significantly higher within TADs than between regions outside of them [18]. While these high-throughput technologies have demonstrated exceptional capabilities in detecting EPIs, they are time-consuming and require substantial investment in sample preparation, library construction, and sequencing equipment.

To address these challenges, numerous computational methods have been proposed to identify EPIs rapidly and accurately on a large scale. These methods primarily utilize two types of data: DNA sequence and epigenetic data. The first category of methods relies entirely on DNA sequence information for EPI prediction. Singh *et al*. [19] introduced a deep learning-based prediction model, SPEID, which integrates convolutional neural networks (CNN) with long short-term memory networks (LSTM). Mao *et al*. [20] proposed a neural network model based on an attention mechanism, EPIANN, which combines attention mechanisms with position-based feature decoding to identify EPIs. Subsequently, Zhuang *et al*. [21] simplified the SPEID model and developed a prediction model called SIMCNN, which employs CNN and transfer learning. The second category involves predicting EPIs using epigenetic data. Roy *et al*. [22] proposed a computational method called RIPPLE, which integrates multiple genomic datasets, including 3C data, chromatin marks, and transcription factor ChIP-seq data. This method employs a random forest and a multi-task learning framework to identify critical datasets and signals for predicting cell line–specific long-range EPIs. Talukder *et al*. [23] developed a method that utilizes various epigenomic features for training, including enhancer RNAs (eRNAs) and four chromatin marks (ChIP-seq data), to predict condition-specific EPIs. Whalen *et al*. [24] introduced TargetFinder, a decision tree–based approach that incorporates measurements such as open chromatin, DNA methylation, gene expression, transcription factors, structural proteins, and modified histone ChIP-seq data. This model helps distinguish true EPI pairs from noninteracting pairs with high precision. Another category combines sequence features and epigenetic data for predicting EPIs. Agarwal *et al*. [25] proposed DeepPHiC, a deep learning framework that integrates multiple features, including sequence information, epigenetic data, and anchor distance, for predicting chromatin interactions. This model demonstrates strong performance in data-scarce scenarios by employing multi-task learning and transfer learning strategies. The TransEPI model [26] utilizes convolutional neural networks (CNNs) and multiple Transformer encoders to capture long-range dependencies, leveraging both sequence features and epigenetic data for EPI prediction. However, its use of multi-layer perceptrons (MLPs) for feature integration fails to fully account for nonlinear representations among features. Most of these computational methods are trained on single-cell-line data, limiting their applicability to identifying cell-line-specific EPIs. When applied to EPIs in other tissues or cell types, the models require retraining. Therefore, developing a global EPI prediction model, enabling a multi-cell-line perspective for comprehensive EPI analysis, is critically needed.

We propose a deep learning framework, KansformerEPI, for predicting EPIs. This framework leverages Kansformer, an encoder based on Kolmogorov–Arnold Networks (KANs) [27] and Transformer [28], to effectively capture nonlinear relationships between features while integrating sequence and epigenetic features. KansformerEPI incorporates CCCTC-binding factor (CTCF) binding sites, DNase-I signals, and five histone modification signals (H3K4me1, H3K4me3, H3K27me3, H3K36me3, and H3K9me3) for EPI prediction while also considering the impact of methylation information. During the experiments, we trained the model using mixed data from two cell lines, GM12878 and HeLa-S3, employing five-fold cross-validation. Independent testing was then conducted on four additional cell lines: HMEC, IMR90, K562, and NHEK.

## Materials and methods

### Datasets

We developed a new model, KansformerEPI, for predicting EPIs. The dataset used in this study is provided by TransEPI and contains enhancer–gene interaction pairs from multiple biological samples. Positive samples in this dataset were identified using 3C methods or genetic approaches, such as expression quantitative trait loci (eQTLs), and are sourced from Benchmark datasets for enhancer–gene interactions (BENGI) [29], representing true enhancer-gene interactions. Negative samples were generated using the following strategy: enhancers that were experimentally measured were paired with genes that have not been experimentally validated to interact with them. The gene selection criterion was that the distance between the gene and the corresponding enhancer should fall within the 95% range of distances observed for positive enhancer–gene pairs. This approach ensures that the negative samples share similar distance characteristics with the positive samples, enabling the model to effectively learn the differences between true interacting pairs and unverified noninteracting pairs.

To predict EPIs, it is first necessary to convert the enhancer-gene interactions identified by Hi-C and ChIA-PET into EPIs. Promoters are defined as regions spanning 1500 bp upstream to 500 bp downstream of the TSS. Using GENCODE annotations, genes in the BENGI dataset were mapped to their corresponding transcripts. Subsequently, low-expression samples (transcripts per million <1) were removed. The detailed information of the final dataset is shown in Table 1, which includes a total of 45 182 positive samples and 307 135 negative samples from six human cell lines (GM12878, HeLa-S3, HMEC, IMR90, K562, and NHEK).

**Table 1:** Summary of the dataset

Cell line	Source	Positive set	Negative set
GM12878	Hi-C	2695	46 212
GM12878	CTCT ChIA-PET	4817	36 028
GM12878	RNAPII ChIA-PET	24,985	70 670
HeLa-S3	Hi-C	2256	21 086
HeLa-S3	CTCT ChIA-PET	1346	10 789
HeLa-S3	RNAPII ChIA-PET	744	2182
HMEC	Hi-C	2286	20 019
IMR90	Hi-C	1468	13 268
K562	Hi-C	2765	73 299
NHEK	Hi-C	1820	13 582

**Figure 1:**
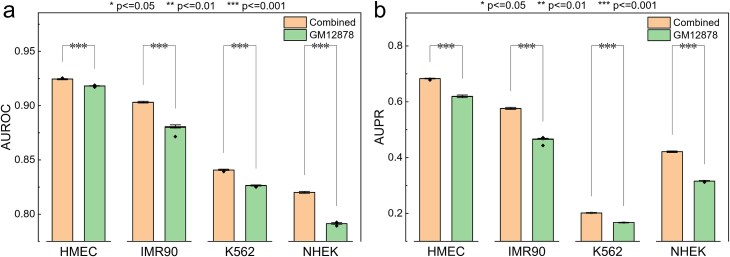
We performed a *t*-test analysis to compare the performance of models trained on the independent GM12878 dataset (GM12878) and models trained on the combined data from two cell lines (combined) across four independent test datasets.

The training set consists of a combination of data from the GM12878 and HeLa-S3 cell lines, containing a total of 36 843 positive samples and 186 967 negative samples. The independent test set is derived from four additional cell lines: HMEC, IMR90, K562, and NHEK, with a total of 8339 positive samples and 120 168 negative samples. The training set was constructed by combining data from the GM12878 and HeLa-S3 cell lines, as referenced in the study by Chen *et al*. Additionally, we trained the model using only the GM12878 cell line, which has a larger number of samples, but found that the performance was inferior to that of the combined training set of the two cell lines. The details are shown in Fig. 1. Furthermore, to assess the impact of data imbalance on EPI prediction, we performed under-sampling to achieve a 1:1 ratio between positive and negative samples.

### Feature extraction

To effectively predict EPIs, we selected eight genomic features based on the chromatin state information of the region, including CTCF binding sites, DNase-I signals, DNA methylation signals, and five histone modification signals (H3K4me1, H3K4me3, H3K27me3, H3K36me3, and H3K9me3). These feature signals are stored in files such as bigwig and narrowPeak formats, which we convert into tensor-stored pt files. In the BENGI dataset, the maximum distance between enhancers and promoters is 2.5 Mbp. Therefore, we predict EPIs within a maximum 2.5 Mbp genomic region. To improve model training efficiency, we divide the 2.5 Mbp genomic region into 5000 consecutive bins, each 500 bp in size, and average the signals within each bin. Additionally, we record the shortest distance from each bin to the enhancer or promoter to enhance the model’s ability to capture E-P pairing positions.

### The KansformerEPI model

#### KansformerEPI framework

**Figure 2:**
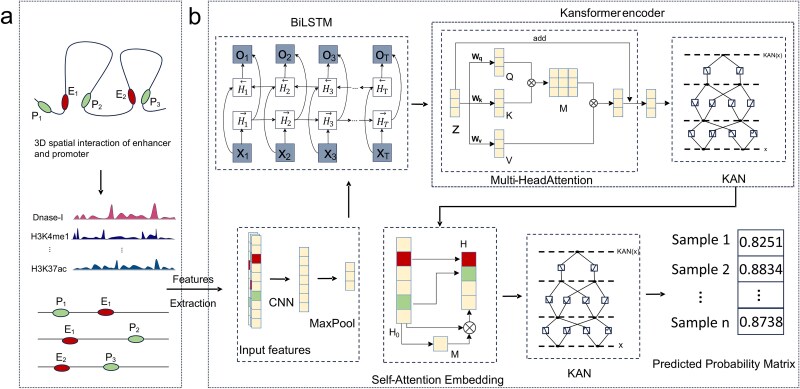
(a) Feature extraction. Features are extracted from the 2.5 Mbp genomic region centered on the enhancer–promoter (E-P) pairs, where red represents enhancers and green represents promoters. (b) KansformerEPI framework.

The architecture of the KansformerEPI model is shown in Fig. 2. First, the KansformerEPI model extracts features from the input signals using a convolutional neural network (CNN). The convolutional operations in the CNN layers effectively identify local patterns and key signal features within the input sequence, providing high-quality feature representations for subsequent layers. Next, the model applies a max pooling layer (MAX Pooling) to downsample the features generated by the convolution, thereby reducing the sequence length, lowering computational complexity, while retaining the main feature signals and reducing noise interference. The downsampled features are then input into a bidirectional long short-term memory (BiLSTM) layer [30]. The BiLSTM processes the input sequence from both forward and backward directions through its two LSTM networks, preserving historical information while incorporating future context, thereby enhancing the model’s ability to capture temporal dependencies.

Subsequently, the output *Z* from the BiLSTM is fed into the Kansformer encoder. This module uses a structure similar to that of the Transformer to capture long-range dependencies. The multi-head self-attention mechanism (MHA) [31] in the encoder captures relationships between features by computing query, key, and value vectors, with the following calculation formula:


$$ Q=Z{W}_q,K={ZW}_k,V={ZW}_v $$

Here, the input sequence $Z\in{R}^{l\times h}$ represents a matrix containing the features of enhancer and promoter loci. ${W}_q$, ${W}_k$, and ${W}_v$ denote three weight matrices used for mapping transformations, while *K*, *Q*, and *V* represent the three feature encodings obtained by applying linear transformations to *Z*. The global relationship matrix *M* is then computed using *K* and *Q*:


$$ M=\mathrm{softmax}\left(\frac{Q{K}^T}{\sqrt{d_k}}\right)V $$

Here, *d* denotes the feature dimension of *Z*. Subsequently, the features in *V* are enhanced using *M*, resulting in the augmented features $\overline{Z}$:


$$ \overline{Z}= MV+b $$

where *b* represents the bias term.

Subsequently, through the KAN layer, the output from the Kansformer encoder is ultimately represented as ${H}_0\in{R}^{l\times h}$. A self-attention embedding method is employed to perform low-dimensional representation learning on ${H}_0$. First, ${H}_0$ is input into a two-layer fully connected network:


$$ {A}_s=\mathrm{softmax}\left({W}_s^2\cdotp \mathit{\tanh}\left({W}_s^1{H}_0^T\right)\right) $$

where ${W}_s^1\in{\mathrm{R}}^{s\times h}$, ${W}_s^2\in{\mathrm{R}}^{r\times s}$, and ${A}_s\in{\mathrm{R}}^{r\times l}$. Each row of the matrix ${A}_s$ sums to 1, representing a set of attention coefficients across all positions in ${H}_0$.

Multiplying ${H}_0$ by ${A}_s$ produces the weighted embedding ${H}_1\in{\mathrm{R}}^{r\times l}$, the mean and maximum values across the second dimension of ${H}_1$ are computed and concatenated with ${H}_1$ to form a low-dimensional embedding. This embedding is then concatenated with the hidden states corresponding to the enhancer and promoter positions, yielding the final input features *H*$\in{R}^{4h}$:


$$ H=\mathrm{AvgPool}\left({H}_1\right)\parallel \mathrm{MaxPool}\left({H}_1\right)\parallel{\mathrm{h}}_e\parallel{\mathrm{h}}_p $$

The aggregated *H* is then fed into the KAN classifier to predict the probability *p* of EPI. To ensure the model is sensitive to the spatial arrangement of enhancers and promoters, the KAN module is also used to predict the distance ${d}_p$ between the enhancer and promoter:


$$ p=\sigma \left({\mathrm{KAN}}_1(H)\right) $$

$$ {d}_p={KAN}_2(H) $$

Here, $\sigma$ denotes the sigmoid activation function, and *p* ranges from 0 to 1, representing the probability of EPI.

Finally, the model is trained using a combination of two loss functions. One is the binary cross-entropy loss ${\mathcal{L}}_{class}$, which is used for predicting EPIs, and the other is the mean squared error loss ${\mathcal{L}}_{dist}$, which is used to predict the distance between the enhancer and the promoter:


$$ {\mathcal{L}}_{\mathrm{class}}\left(y,p\right)=-\frac{1}{N}\sum_i^N\kern0.1em \left[{y}_i\log \left({p}_i\right)+\left(1-{y}_i\right)\log \left(1-{p}_i\right)\right] $$

$$ {\mathcal{L}}_{dist}\left({d}_p,{d}_t\right)=\frac{1}{N}\sum_i^N\kern0.1em {\left({d}_{p,i}-{d}_{t,i}\right)}^2 $$

$$ \mathcal{L}={\mathcal{L}}_{class}+{\mathcal{L}}_{dist} $$

Here, $\mathcal{L}$ represents the total loss function, ${y}_i$ denotes the ground truth label (0 or 1) for the *i*-th sample, ${p}_i$ indicates the predicted probability, ${d}_p$ represents the predicted enhancer–promoter distance, and ${d}_t$ corresponds to the true distance.

#### Kansformer encoder

In deep learning models, the MLP serves as a fundamental building block and is commonly used in machine learning to approximate nonlinear functions. However, when integrating features, MLPs do not fully account for nonlinear transformations between features. To address this limitation, Liu *et al*. proposed a novel architecture, Kolmogorov–Arnold Network (KAN), which, while structurally similar to MLPs, is based on the Kolmogorov–Arnold representation theorem and offers distinct advantages in capturing feature nonlinearity. The primary difference between KAN and MLP lies in their activation function mechanisms: MLPs employ fixed activation functions at nodes (neurons), whereas KAN introduces learnable activation functions through its weights, thereby enhancing the model’s flexibility and adaptability. A K-layer KAN network can be represented as a nested composition of multiple KAN units:


$$ \mathrm{KAN}(Z)=\left({\varPhi}_{K-1}\circ{\varPhi}_{K-2}\circ \cdots \circ{\varPhi}_1\circ{\varPhi}_0\right)(Z) $$

where ${\varPhi}_i$ denotes the *i*-th layer of the KAN network. For each KAN layer, the input dimension is ${n}_{in}$, and the output dimension is ${n}_{out}$. The definition of a KAN layer can be expressed as follows:


$$ \varPhi =\left\{{\varphi}_{k,q,p}\right\},p=1,2,\dots, {n}_{\mathrm{in}},q=1,2,\dots, {n}_{\mathrm{out}} $$

The activation process of each KAN layer consists of two steps: first, the input ${Z}_{k,p}$ is transformed using the activation function ${\varphi}_{k,q,p}$, yielding the post-activation value ${\overset{\sim }{Z}}_{k,q,p}$. Then, the activation values of all inputs are summed to produce the neuron’s activation value:


$$ {Z}_{k+1,q}=\sum_{p=1}^{n_k}\kern0.1em {\overset{\sim }{Z}}_{k,q,p}=\sum_{p=1}^{n_k}\kern0.1em {\varphi}_{k,q,p}\left({Z}_{k,p}\right) $$

Leveraging the advantageous properties of KAN, the Kansformer encoder architecture was proposed, replacing the MLP layers in Transformer encoders with KAN layers to better handle the classification task of EPIs. The structure of the Kansformer encoder is shown in Fig. 2, featuring an alternating stack of MHA layers and KAN layers. Layer normalization is applied before each module, and residual connections are used after each module. This design not only captures both local and global dependencies within sequences but also integrates the nonlinear transformations of features through the KAN layers, enabling more effective feature extraction. By overcoming the limitations of traditional MLPs, this architecture significantly improves the prediction of EPIs.

### Parameter configuration

The training environment for KansformerEPI utilized an NVIDIA A100 GPU with 80 GB of memory, and the Python version was 3.9.18. The number of training epochs was set to 300; however, an early stopping criterion was implemented. Training terminated early if the model’s performance on the validation set, measured as the mean of AUROC and AUPR, showed no improvement over several consecutive epochs, as specified by the patience parameter (patience = 10). The convolutional layer is configured with 180 channels, a kernel size of 11, and a pooling size of 10. The model employs a three-layer encoder, with each layer utilizing six attention heads and a feedforward neural network hidden layer dimension of 256. Each model includes two fully connected layers with output dimensions of 128 and 64, respectively, followed by a dropout rate of 0.2. The AdamW optimizer was employed to update the trainable weights of the neural network, with the learning rate set to 0.0001. The loss function was a combination of binary cross-entropy loss (BCELoss) and mean squared error loss (MSELoss). Training was conducted using five-fold cross-validation, where each epoch was trained on four folds and validated on the remaining fold.

### Evaluation metrics

To evaluate the performance of the KansformerEPI model in predicting EPIs, we employed two commonly used evaluation metrics: the area under the receiver operating characteristic curve (AUC) and the area under the precision–recall curve (AUPR).

The AUC measures the model’s ability to distinguish between positive and negative samples and represents the area under the receiver operating characteristic (ROC) curve. The ROC curve is formed by plotting the true positive rate (TPR) and false positive rate (FPR) at various threshold values:

The relevant formula for TPR is as follows:


$$ TPR=\frac{TP}{TP+ FN} $$

where *TP* represents the number of true positives, and *FN* represents the number of false negatives.


$$ FPR=\frac{FP}{FP+ TN} $$

where *FP* represents the number of false positives, and *TN* represents the number of true negatives.

The AUC value ranges from 0 to 1, with higher values indicating better model performance. An AUC close to 1 signifies that the model can almost perfectly distinguish between positive and negative samples, while an AUC close to 0.5 indicates that the model’s performance is similar to random guessing.

AUPR is used to evaluate the model’s performance on highly imbalanced datasets, particularly when the number of positive samples is much smaller than that of negative samples in prediction tasks. The AUPR measures the model’s ability to capture positive samples at different thresholds by calculating the area under the precision–recall (PR) curve. The PR curve is composed of precision and recall, defined by the following formulas:

Precision:


$$ Precision=\frac{TP}{TP+ FP} $$

represents the proportion of correctly predicted positive samples among all predicted positive samples.

Recall:


$$ Recall=\frac{TP}{TP+ FN} $$

represents the proportion of correctly predicted positive samples among all actual positive samples.

The higher the AUPR value, the better the model maintains high precision at higher recall rates. The AUPR is particularly suited for evaluating imbalanced datasets, as it emphasizes the model’s performance when the number of positive samples is low.

## Results

### Comparison of KansformerEPI with existing methods

To comprehensively evaluate the effectiveness of our prediction method, we compared KansformerEPI with five state-of-the-art deep learning models (TransEPI, TargetFinder, SPEID, 3DPredictor, and DeepTACT). We trained all models using the same chromosome segmentation scheme to eliminate potential errors introduced by differences in data splitting. The GM12878 and HeLa-S3 datasets were used for training, while the HMEC, IMR90, K562, and NHEK datasets were used for testing. We ran 30 experiments on each cell line and conducted identical training and testing procedures for the TransEPI model according to the model parameters provided.

To further evaluate the predictive performance of the KansformerEPI model, we compared it with the existing TransEPI model and performed a two-sample *t*-test to analyze the mean differences between the two methods across four cell lines. A significance level of 0.05 was set, and if the *P*-value was <.05, it was considered that the two methods exhibited a significant difference in their predictive performance for the respective cell lines; otherwise, if the *P*-value was >.05, the difference was considered nonsignificant.

**Figure 3:**
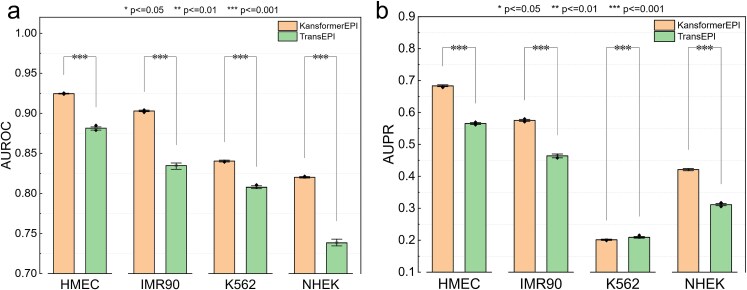
A two-sample *t*-test was conducted on the performance of the KansformerEPI and TransEPI models across the four cell lines, followed by a comparative analysis of the AUROC and AUPR metrics.

**Figure 4:**
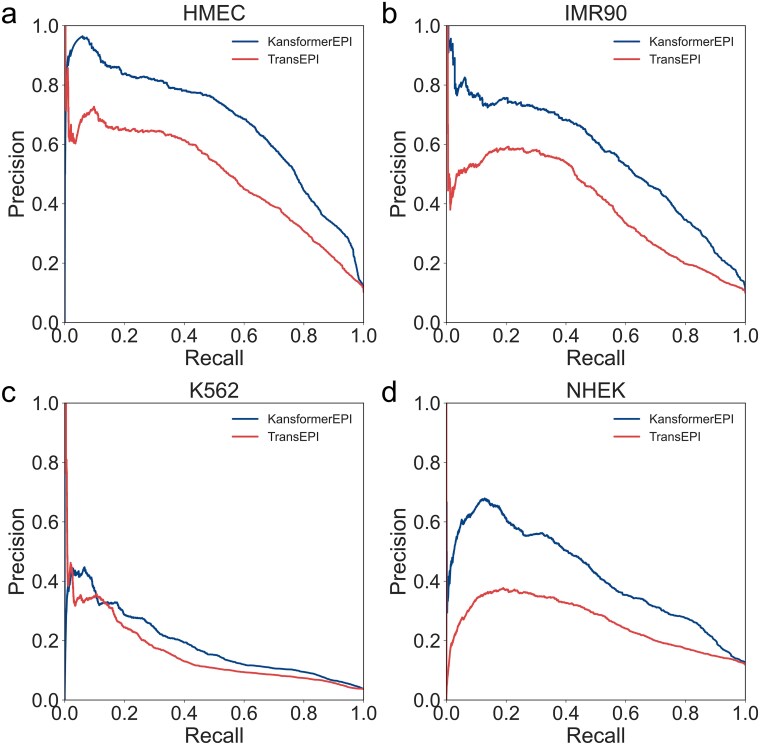
The precision–recall curves (PRCs) of KansformerEPI and TransEPI on four independent test datasets.

As shown in Fig. 3a, the AUC differences between the two methods across the four cell lines are presented. The results show that our KansformerEPI method outperforms TransEPI in all four cell lines, with statistically significant differences. Notably, in the IMR90 and NHEK cell lines, the AUC improvement with KansformerEPI is ~7% and 8%, while the improvement is ~4% in the HMEC and K562 cell lines. Figures 3b and 4 show the AUPR differences between the two methods across the four cell lines. While the results are comparable in the K562 cell line, significant differences were observed in the HMEC, IMR90, and NHEK cell lines, with a clear improvement in performance.

Similarly, Table 2 presents the performance comparison between our model and other existing models in terms of AUROC and AUPR. In terms of AUROC, our model improves upon the best-performing model in the HMEC, IMR90, and K562 cell lines by 0.54%, 5.7%, and 3.3%, respectively. However, in the NHEK cell line, our model performs 2.7% worse than the best model. In terms of AUPR, we find that in the K562 cell line, our model performs comparably to the best model; while in the HMEC, IMR90, and NHEK cell lines, our model outperforms the second-best model, with improvements of 11.7%, 11.1%, and 4.2%, respectively. Overall, although there is a slight gap in the NHEK cell line, our model outperforms other existing models in most cell lines.

**Table 2:** A performance comparison of KansformerEPI with other existing models in terms of AUROC and AUPR metrics

	Dataset	KansformerEPI	TransEPI	TargetFinder	SPEID	3DPredictor	DeepTACT
AUROC	HMEC	0.9246	0.8815	0.8671	0.4939	0.9192	0.5480
	IMR90	0.9030	0.8348	0.8174	0.4909	0.8459	0.5487
	K562	0.8405	0.8078	0.8000	0.4909	0.8047	0.4784
	NHEK	0.8202	0.7385	0.7942	0.4954	0.8476	0.5681
AUPR	HMEC	0.6832	0.5654	0.4488	0.1019	0.5150	0.1358
	IMR90	0.5751	0.4641	0.3850	0.0962	0.3975	0.1263
	K562	0.2013	0.2092	0.1252	0.0361	0.1601	0.0345
	NHEK	0.4210	0.3114	0.3138	0.1165	0.3787	0.1749

### The impact of methylation on enhancer–promoter interaction prediction

**Figure 5:**
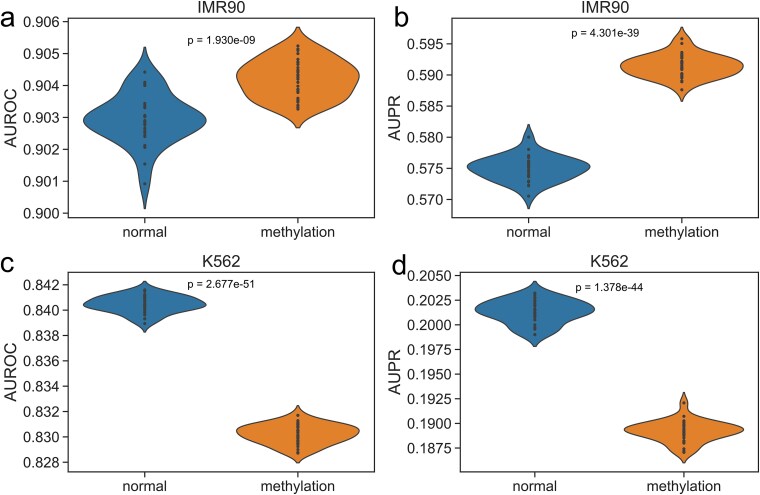
After incorporating methylation features, a two-sample *t*-test analysis was conducted on the AUROC and AUPR metrics to observe the impact on EPI prediction.

In our experiments, we incorporated DNA methylation data based on CTCF binding sites, DNase-I signals, and five histone modification signals. After extracting methylation signals, we performed normalization for all features. We referenced the study by Whalen *et al*. [24] to obtain methylation feature sets for four cell lines: GM12878, HeLa-S3, IMR90, and K562, selecting these four cell lines as datasets to investigate the impact of methylation on EPI prediction results. For training and testing, we followed the previous scheme, merging data from GM12878 and HeLa-S3 to construct the training set, and then independently tested the model on IMR90 and K562. During testing, we conducted 30 rounds of experiments, and the results are shown in Fig. 5.

In the IMR90 dataset, the AUROC metric was similar, but the AUPR was ~1.67% higher. In the K562 dataset, both AUROC and AUPR were ~1% lower. Although methylation showed a slight enhancement effect on EPI prediction in some datasets, it produced opposite results in others. This variation suggests that the role of methylation in EPIs prediction may not be universally applicable. Currently, we only have methylation data from these two sets for testing, so we lack sufficient evidence to demonstrate a significant promotion effect of methylation on EPIs prediction. While some studies suggest that methylation contributes positively to EPI prediction [24], we did not observe this phenomenon in our experiments.

### Analysis of the impact of dataset balance on enhancer–promoter interaction prediction

In the task of EPI prediction, the original dataset is highly imbalanced, with some datasets having a ratio of 1:20 between positive and negative samples. The positive samples are experimentally determined, while the negative samples are generated by pairing enhancers that do not interact with genes. To assess the impact of dataset balance on EPI prediction, this study employed an undersampling strategy, adjusting the dataset to a 1:1 ratio of positive to negative samples. It is believed that using a balanced 1:1 dataset can effectively mitigate the model’s class imbalance bias. In imbalanced datasets, models may favor the majority class (negative samples), whereas a balanced dataset helps the model focus more on positive samples, thereby improving its ability to identify positive interactions. This data balancing strategy contributes to the stability of the model’s performance on positive samples.

**Figure 6:**
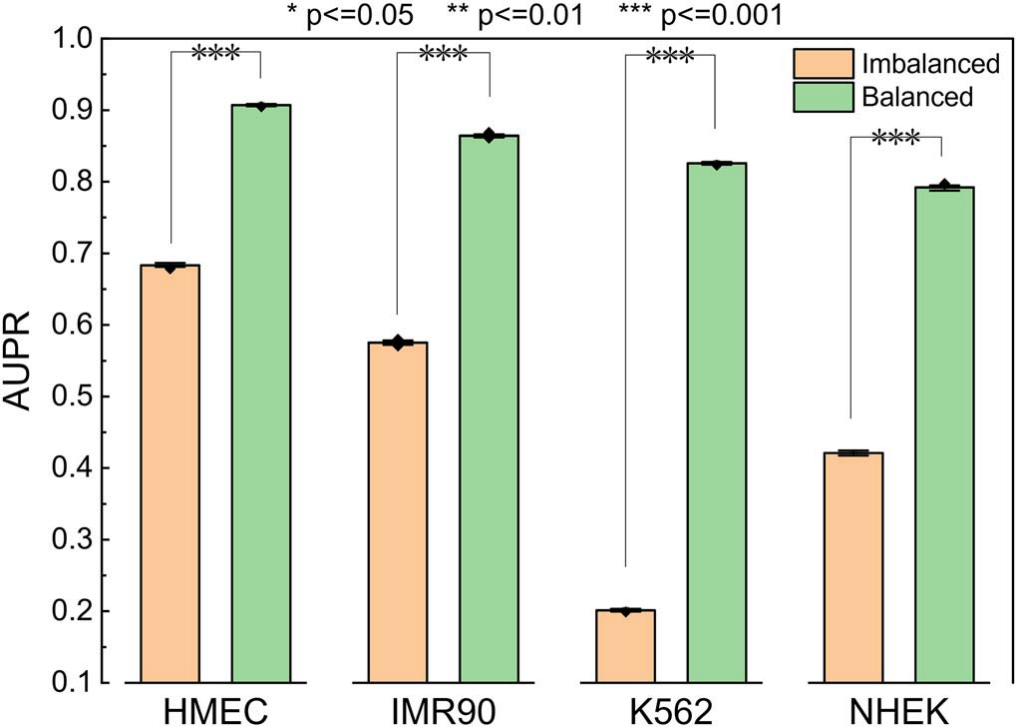
The model trained on the balanced dataset was tested on both balanced and imbalanced test sets, and a two-sample *t*-test was used to analyze the differences in the AUPR metric. “Imbalanced” refers to the imbalanced dataset, while “balanced” refers to the balanced dataset.

**Figure 7:**
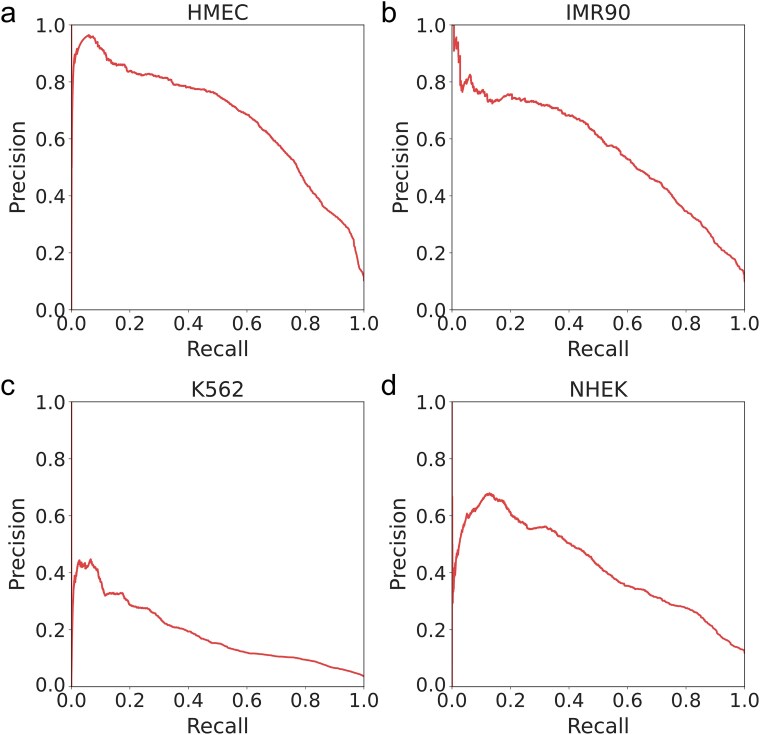
Precision–recall curves for a model trained on a balanced dataset and tested on four imbalanced cell lines.

As shown in Fig. 6, we retrained the model using a balanced dataset. Subsequently, we evaluated its performance on two test sets: one consisting of imbalanced cell lines and another processed with undersampling. Each test was repeated 30 times, and at-test analysis was conducted to assess statistical significance. For the imbalanced cell line test, results were visualized using a PR curve, as depicted in Fig. 7. Our findings indicate that the model trained on balanced datasets consistently outperformed those trained on imbalanced data, with statistically significant improvements. Therefore, we conclude that balancing the training data enhances model performance.

### Feature importance analysis of enhancer–promoter interactions

**Figure 8:**
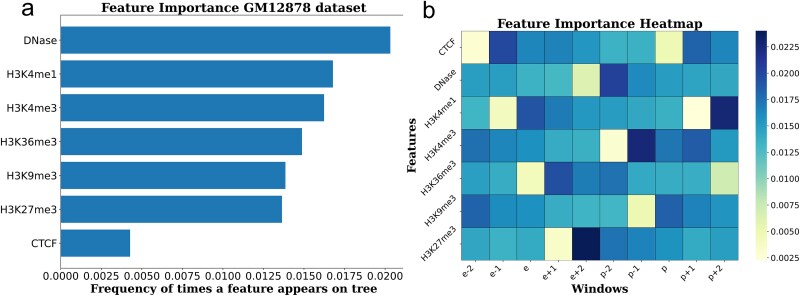
The terms e − 2, e − 1, e + 1, and e + 2 represent windows near the enhancer, while p − 2, p − 1, p + 1, and p + 2 represent windows near the promoter. (a) Importance analysis of the seven features in the GM12878 cell line. (b) Selection of the more important windows near enhancers and promoters, with a window size of 500 bp, followed by a window-based importance analysis of the features.

In this study, we utilized the random forest model to conduct an in-depth analysis of the key features for EPIs, focusing on the importance of features and their contributions within different windows. To achieve this, we first computed the importance scores for each feature across multiple windows to assess its impact on EPI prediction. To ensure the systematic nature of the analysis, we selected the most important windows for enhancers and promoters from 5000 windows and calculated the contribution of seven features in each window. Through this window-based analysis, we were able to observe the variations in feature importance across different gene regulatory regions, thus identifying which features have a significant impact on EPI prediction within specific windows. As shown in Fig. 8a, we computed the importance scores of these features within the selected windows and averaged them to determine the relative importance of the seven features in the GM12878 cell line. The results indicate that the DNase-I signal had the highest score, suggesting its most prominent role in prediction. In contrast, the CTCF binding sites had the lowest score, while H3K4me1 and H3K4me3 exhibited relatively high importance. This could be due to the fact that these two features are commonly found in enhancer regions, which tend to be more active in EPI prediction.

Subsequently, we visualized the distribution of feature importance scores within each window using heatmaps. As shown in Fig. 8b, the heatmap displays the differences in the importance scores of the seven features (including CTCF, DNase-I, and five histone modification signals) across different windows. The color intensity in the heatmap reflects the level of feature importance: darker colors indicate higher importance for predicting EPIs, while lighter colors represent lower importance. Through heatmap analysis, we can intuitively understand the contribution of each feature across various genomic regions. The terms e and p represent the enhancer and promoter windows, respectively, while windows such as e-1 and p-1 represent those adjacent to them. Within the enhancer windows, we observe that H3K4me1 has the darkest color, highlighting its prominent role in the enhancer regions. In windows adjacent to the enhancer, we find that H3K9me3, H3K27me3, and CTCF have higher importance scores. Similarly, within the promoter window, H3K9me3 exhibits the highest color intensity, indicating its greater significance. In windows near the promoter, H3K4me1 and H3K4me3 show higher importance. This observation provides new biological insights into the regulatory mechanisms of EPIs and further validates the key roles of these features in gene regulation. Moreover, these findings offer valuable references for subsequent biological studies, helping us better understand EPIs and their biological functions.

**Table 3:** Statistical distribution of the four regulatory relationships

Dataset	Single	MO	MI	MIMO	Total
HMEC	38	316	2010	265	2285
IMR90	59	390	1221	367	1468
K562	44	1327	2420	1346	2765
NHEK	28	372	1712	354	1820

**Figure 9:**
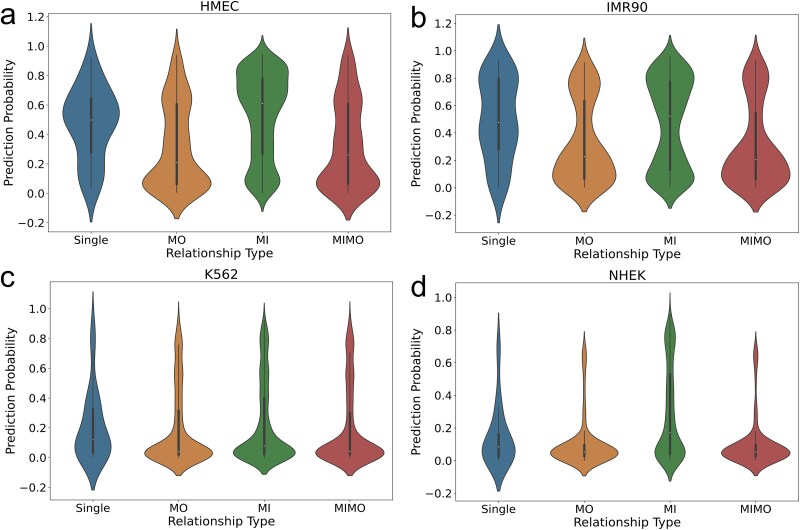
Prediction probability distribution of the four EPI patterns across four cell lines.

### Analysis of enhancer–promoter interaction pattern classification and prediction performance

EPIs typically form subnetworks of various biological processes. To better analyze and understand these interaction patterns, we computed the number of promoters regulated by each enhancer (outdegree) and the number of enhancers regulating each promoter (indegree). In our data, on average, each enhancer regulates one to two promoters, while each promoter is regulated by two to three enhancers. Inspired by the work of Sushmita Roy *et al*. [22], we classified the connection patterns between enhancers and promoters into the following four categories: (i) Single Interaction: a one-to-one relationship, where one enhancer interacts with only one promoter; (ii) Multi-output Component (MO): a single enhancer regulates multiple promoters; (iii) Multi-input Component (MI): a single promoter is regulated by multiple enhancers; (iv) Multi-input Multi-output Component (MIMO): multiple enhancers regulate multiple promoters, forming a network.

When analyzing the four test cell lines, we counted the occurrences of these four regulatory relationships and the predicted probabilities from our model. The results show that among these four regulatory relationships, MI accounts for the largest proportion, as shown in Table 3. The largest proportion of this regulatory pattern in MI may be due to the following reasons: when analyzing the number of promoters and enhancers, humans have over 20 000 genes. According to the EnhancerAtlas [32] database, we have gathered information on a total of 4 506 217 human enhancers. The number of enhancers is much higher than that of promoters, and this imbalance in their quantities could result in a higher proportion of regulation where a single promoter is controlled by multiple enhancers. The phenomenon observed in our experiments coincides with biological insights in this regard. Additionally, the box plots in Fig. 9 reveal the distribution of predicted probabilities for the four regulatory relationships, with MI consistently exhibiting the highest prediction accuracy. This result indicates that in the BENGI dataset, the MI relationship not only dominates in terms of quantity but also demonstrates the best prediction accuracy. Our model’s predictive power stems from replacing traditional linear layers in MLP with KAN nonlinear layers. This allows precise capture of nonlinear synergies or competitions among multiple enhancers, making it better suited for multi-enhancer regulation scenarios. As a result, the model demonstrates greater biological plausibility in complex multi-enhancer regulation predictions.

### The DKK1 gene inhibits the migration and invasion of breast cancer cells

The DKK1 (Dickkopf-related protein 1) gene is located on human chromosome 10 and encodes a secreted glycoprotein belonging to the Dickkopf protein family. Its primary function is to inhibit the canonical Wnt/β-catenin signaling pathway by forming a complex with the receptors LRP5/6 and Kremen proteins, thereby regulating processes such as embryonic development, cell differentiation, bone formation, and tumorigenesis [33]. Due to the critical role of the DKK1 gene, numerous studies have investigated its involvement in disease. For instance, Yu *et al*. [34] explored the high-expression characteristics of DKK1 in hepatocellular carcinoma and its relationship with carcinogenic mechanisms.

In the HMEC cell line, we evaluated and compared the predictive performance of our model and TransEPI on EPIs associated with the DKK1 gene. The results demonstrate that our model achieved higher interaction probability scores when identifying experimentally supported DKK1-related EPIs. Specifically, we examined four enhancers, EH37E0171937, EH37E0171940, EH37E0171907, and EH37E0171908, that have been experimentally validated as interacting with DKK1. These enhancer–gene interactions were obtained from the BENGI dataset, which integrates data from the cCRE Registry and experimentally derived genomic interaction datasets. The BENGI annotations infer enhancer–gene interactions based on three types of experimental evidence: (i) 3D chromatin interaction data (ChIA-PET, Hi-C, and CHi-C), used to identify enhancer–promoter pairs that exhibit physical proximity within ±2 kb of the TSS; (ii) genetic interaction data (*cis*-eQTL), which associate enhancer regions with SNPs linked to gene expression changes; and (iii) functional perturbation assays (crisprQTL using CRISPR/dCas9), which validate the regulatory impact of enhancers on target gene expression [29].

We compared the interaction probability scores predicted by our model and by TransEPI for these four EPIs. Both models generate scores ranging from 0 to 1, with higher values indicating greater confidence in the model’s prediction of an EPI. As shown in Table 4, the predicted probabilities for the four enhancer–DKK1 interactions are presented, where “Yes” denotes experimentally validated regulatory relationships according to the BENGI dataset. Our model achieved a maximum predicted interaction probability of 0.9453 and a minimum of 0.9259, whereas other models such as TransEPI yielded average probabilities of ~0.42. These results clearly indicate that our model exhibits significantly superior predictive performance for DKK1-associated EPIs. Furthermore, the connection patterns of these EPIs are primarily of the MI type. Additionally, research by Gao *et al*. [35] has demonstrated that DKK1 inhibits the migration and invasion of breast cancer cells by suppressing the expression of β-catenin and MMP7, thereby acting as a tumor suppressor in breast cancer, and that abnormal expression of the DKK1 gene may lead to tumorigenesis. The application of our model thus provides a novel perspective for identifying potential causes of DKK1 dysregulation from the enhancer standpoint, offering computational biology evidence to support biologists in elucidating the mechanisms underlying disease pathogenesis.

**Table 4:** Performance comparison on four validated DKK1-associated EPIs

Enhancer	Gene	Kansformer	TransEPI	Experiment evidence
EH37E0171937	ENSG00000107984.5	0.9453	0.3970	Yes
EH37E0171940	ENSG00000107984.5	0.9451	0.3804	Yes
EH37E0171907	ENSG00000107984.5	0.9329	0.4384	Yes
EH37E0171908	ENSG00000107984.5	0.9259	0.4520	Yes

## Conclusion

In this study, we propose a method, KansformerEPI, which integrates a KAN and Transformer-based encoder to predict EPIs using both sequence and epigenetic features. Compared to existing models, our approach leverages KAN to effectively capture the nonlinear relationships between features during feature integration, strengthening the important connections between features and thus improving the accuracy of EPI predictions. Most existing methods use EPI prediction models built on single cell lines, which limits their ability to identify EPI relationships that are specific to the respective cell line. To enable EPI prediction across different tissues, we adopted five-fold cross-validation with chromosomal partitioning, using GM12878 and HeLa-S3 cell lines for training, and tested the model performance on four independent cell line datasets following the same chromosomal partitioning scheme. Through this approach, we constructed a global EPI prediction model, KansformerEPI.

Furthermore, existing methods rarely consider the impact of methylation features on EPI prediction, despite the critical role of DNA methylation in gene regulation. Therefore, we incorporated methylation features into our model and trained the model on the GM12878 and HeLa-S3 cell lines, subsequently testing it on two independent cell line datasets to evaluate the influence of methylation on EPI prediction. We observed that methylation had a significant positive effect on EPI prediction in the IMR90 cell line, but its impact was less pronounced in the K562 dataset. While some studies suggest that methylation enhances EPI prediction, we did not observe this effect in our experiments. This may be attributed to potential issues within the K562 dataset, as its performance in predicting EPIs was noticeably lower than other cell lines even without methylation features. Subsequent analysis of interaction models for positive samples revealed that the most prevalent pattern is MI, where a promoter is regulated by multiple enhancers simultaneously. By substituting linear layers with KAN nonlinear layers in our model improvement, we can more accurately capture the nonlinear synergies or competitions among multiple enhancers. This enhancement enables better EPI prediction.

In summary, our model, KansformerEPI, which integrates KAN and Transformer, has demonstrated excellent performance in the global analysis of EPIs across multiple cell lines. However, the self-attention mechanism in Transformer models requires the storage of relationships between each sequence element and all other elements, which is highly memory-intensive. Additionally, KAN’s training speed is relatively slow; compared to MLPs with the same number of parameters, KAN typically requires up to 10 times longer for training. To address these challenges, future research will focus on improving the training efficiency of KAN and seeking innovative solutions to reduce the memory consumption of Transformers.

**Key Points**

- KansformerEPI integrates KAN and Transformer to address the limitations of traditional methods that inadequately consider nonlinear relationships among features. These nonlinear relationships are critically important for accurate prediction of EPIs. By leveraging KAN, KansformerEPI effectively captures the complex interactions between diverse epigenetic and sequence features, thereby enabling more precise EPI predictions.
- KansformerEPI facilitates a global analysis of EPIs across multiple cell lines, eliminating the complexity of generating a separate training model for each tissue. Using a unified model, it enables cross-tissue prediction of EPIs for different cell types, enhancing model scalability and avoiding the redundancy of designing single-tissue models. This approach reduces dependency on extensive data, making the model more adaptable to various tissues.
- Through the analysis of EPI networks across multiple cell lines, we found that a single promoter regulated by multiple enhancers predominates among various types of regulatory relationships. Moreover, this type of regulatory interaction exhibits higher reliability.

## Data Availability

The datasets and codes are available at https://github.com/shaohongyihhh/KansformerEPI.
